# Case Report: Oral Fecal Microbiota Transplantation in a Dog Suffering From Relapsing Chronic Diarrhea—Clinical Outcome and Follow-Up

**DOI:** 10.3389/fvets.2022.893342

**Published:** 2022-07-04

**Authors:** Matteo Cerquetella, Andrea Marchegiani, Giacomo Rossi, Massimo Trabalza-Marinucci, Fabrizio Passamonti, Marco Isidori, Fabrizio Rueca

**Affiliations:** ^1^School of Biosciences and Veterinary Medicine, University of Camerino, Camerino, Italy; ^2^Department of Veterinary Medicine, University of Perugia, Perugia, Italy

**Keywords:** case report, dog, fecal microbiota transplantation, chronic diarrhea, oral capsules

## Abstract

The present case report describes the effects of orally administered fecal microbiota transplantation (FMT) (frozen capsules) in a dog suffering from relapsing chronic diarrhea, needing a continuous low prednisolone dose to maintain the condition under acceptable control. Through FMT, we aimed at evaluating the possibility of improving the clinical score and/or reducing/suspending steroid administration. During a first period of strict monitoring (21 days), the canine inflammatory bowel disease activity index (CIBDAI) score passed from mild to clinically insignificant disease. Furthermore, two additional gastrointestinal signs that had been reported, bloating and episodes of painful defecation, rapidly improved (bloating) or even resolved (painful defecation). The patient was then followed for 18 months (to the authors' knowledge, the longest follow-up time ever reported in a dog), during which no serious relapses occurred and no increase in prednisolone dose was necessary. No adverse clinical effects were ever reported during monitoring. The present description provides a further experience increasing those already present in the veterinary literature, in which an agreement on how to use FMT has not yet been achieved although strongly needed and recommended.

## Introduction

Fecal microbiota transplantation is considered a promising way to rebuild the intestinal environment and possibly treat some gastrointestinal (GI) and non-GI diseases. Nevertheless, although widely studied, there are many points that need to be clarified and on which widespread agreement is lacking (1). For example, if we look at the specific mechanism of action of fecal microbiota transplantation (FMT), it is very likely that this is not unique, but that it can be effective in different ways, also depending on the condition to be restored/treated (1). FMT can increase bacterial diversity, provide bacteriocins and bacteriophages, and can also drive nutrient metabolism, including primary bile acids' conversion. Furthermore, by possibly re-establishing eubiosis, it can promote a healthy and functional gut barrier and a healthy and functional immune system (2). In human medicine, it has been and is increasingly studied for many different conditions such as, for example, chronic enteropathies (e.g., inflammatory bowel disease [IBD], irritable bowel syndrome), liver diseases, obesity, metabolic syndrome, and neuropsychiatric disorders. However, its most widely recognized indication is the recurrent *Clostridium difficile* infection refractory to standard therapy (2). Another fundamental aspect, on which an international agreement is not present, is how FMT should be considered and, consequently, legally regulated. Variably depending on different countries, FMT can be considered, for example, as a biological agent or a medicinal product or, like in Italy, as a transplant of cells/tissues (1, 3). Finally, although it is considered a generally safe procedure, potential short and, especially, mid- and long-term risks possibly associated with FMT still need to be fully investigated (1, 4).

Similarly, and with a certain delay regarding human medicine, a clear consensus is also lacking in veterinary medicine, and no universally recognized guidelines are present in the veterinary field (strongly needed) (5). Nevertheless, a recent review paper provides interesting updates and recommendations on FMT use (6). If we look at veterinary literature (dogs and cats), only a few clinical studies (including case reports and case series) have been published, compared to human ones. More precisely, to the best of our knowledge, six case reports (1 as abstract proceedings) (7–12) and 12 clinical studies/case series (6 of which as abstracts) (13–24) are reported in the literature, plus one commentary (25), one review (6), and one observational study (5), in addition to other articles not only focused on canine and feline FMT but also referring to it at different levels [for example, (26, 27)]. Considering variables such as study design, donor and recipient selection, number of patients included, species (dog vs. cat), disease treated, route of administration, dosage (how much, for how many days, etc.,), and follow-up, although providing invaluable information, such literature needs to be enriched.

In this case report, we describe the case of a dog suffering from relapsing chronic diarrhea, needing a continuous low prednisolone dose to maintain the condition under acceptable control, and nevertheless presenting periodic relapses, managed with oral FMT to evaluate the possibility of improving the clinical score and/or reducing/suspending prednisolone administration. Our aim is to furnish a further experience to be added to those already present in order to provide additional evidence, possibly useful to define when/how/why to use FMT in veterinary medicine.

## Clinical Case Description

### Patient History and Clinical Presentation

The case described is that of a male Labrador dog, 6 years old at the time of FMT. The patient had a previous history of partially-/un-controlled chronic diarrhea (the management included different antimicrobials' courses, dietary changes, budesonide, etc.) due to IBD (lymphoplasmacytic enteritis); the diagnosis confirmed as reported in the literature (28) at the time of presentation (around 2 years before FMT). In the couple of years following initial presentation and preceding FMT, the dog had been under acceptable control with cycles of psyllium husk and probiotics, a hydrolysates diet, and prednisolone tapered to a minimum effective dose ranging from 0.08 to 0.16 mg/kg/day. Sporadic clinical relapses were successfully managed by increasing prednisolone for short periods and temporarily modifying the diet (homemade single protein/fiber source). At the time of FMT administration, the patient was presenting a canine IBD activity index (CIBDAI) score (29) of four (mild IBD), mainly due to problems related to stool consistency and frequency. The dog was also presenting recurrent bloating and episodes of painful defecation. The decision to make an attempt with FMT was taken to evaluate the possibility of improving the clinical score and/or decrease bloating and painful defecation and/or reduce/suspend prednisolone administration.

### FMT Protocol and Controls

A female intact Argentine Dogo was selected as the fecal donor according to a stringent screening protocol developed from previously published guidelines (25), in an attempt to avoid the transmission of infectious diseases, as well as maximizing the chances of infusion of a desirable fecal microbiome and metabolome. Positive screening criteria included age range of 1–8 years; regular core vaccination schedules; no history of raw-feeding and dietary indiscretion; no history of chronic GI diseases and other chronic disorders (e.g., neoplastic, immune mediated, and endocrine); no history of antimicrobial administration in the last 6 months; having a good nutritional status (i.e. scores of 4 and 5 on a 9-point body condition scale, with no loss of muscle mass); normal fecal consistency; and deemed healthy on physical examination. Furthermore, the candidate was required to have unremarkable complete blood count and extended serum biochemistry panel and urinalysis. Lastly, negative testing for the following coprological examinations was verified: centrifugal fecal flotation with zinc sulfate solution for the detection of helminth ovas; immunofluorescence assay for *Giardia duodenalis* cysts and *Cryptosporidium* spp. oocysts executed on samples collected on three consecutive days; fecal culture for the detection of *Salmonella* spp. and *Campylobacter* spp.; quantitative PCR for the detection of canine enteric coronavirus and distemper virus.

Fecal microbiota transplantation capsules were manufactured following the protocol developed by Hirsch et al. (30), with adaptations. Briefly, an amount of 100 g (wet weight) of freshly voided feces was homogenized in 0.9% NaCl solution, sieved through a fine mesh strainer, and spun twice at different rotational speeds in order to remove particulate matter and concentrate the microbial fraction. The obtained pellet was then re-suspended in 0.9% NaCl, combined with glycerol (15% v/v) as a cryoprotectant and pipetted into gastro-resistant size 0 capsules, which were closed and then secondarily sealed in size 00 gastro-resistant capsules. Each capsule contained 650 μl of fecal slurry, corresponding to a (mean) total bacterial count of 8.6 (±0.8) x 10^11^ colony forming units (CFUs) and (mean) total coliform count of 2.6 (± 1.3) x 10^9^ CFUs. Bacterial quantifications were performed by the pour plate method, averaging the CFU counts of three technical replicates. Batches of stool capsules were stored frozen at −80°C and were used within 12 months from preparation in order to assure a satisfactory microbiota similarity with that of the fresh samples (31).

**Figure 1:**
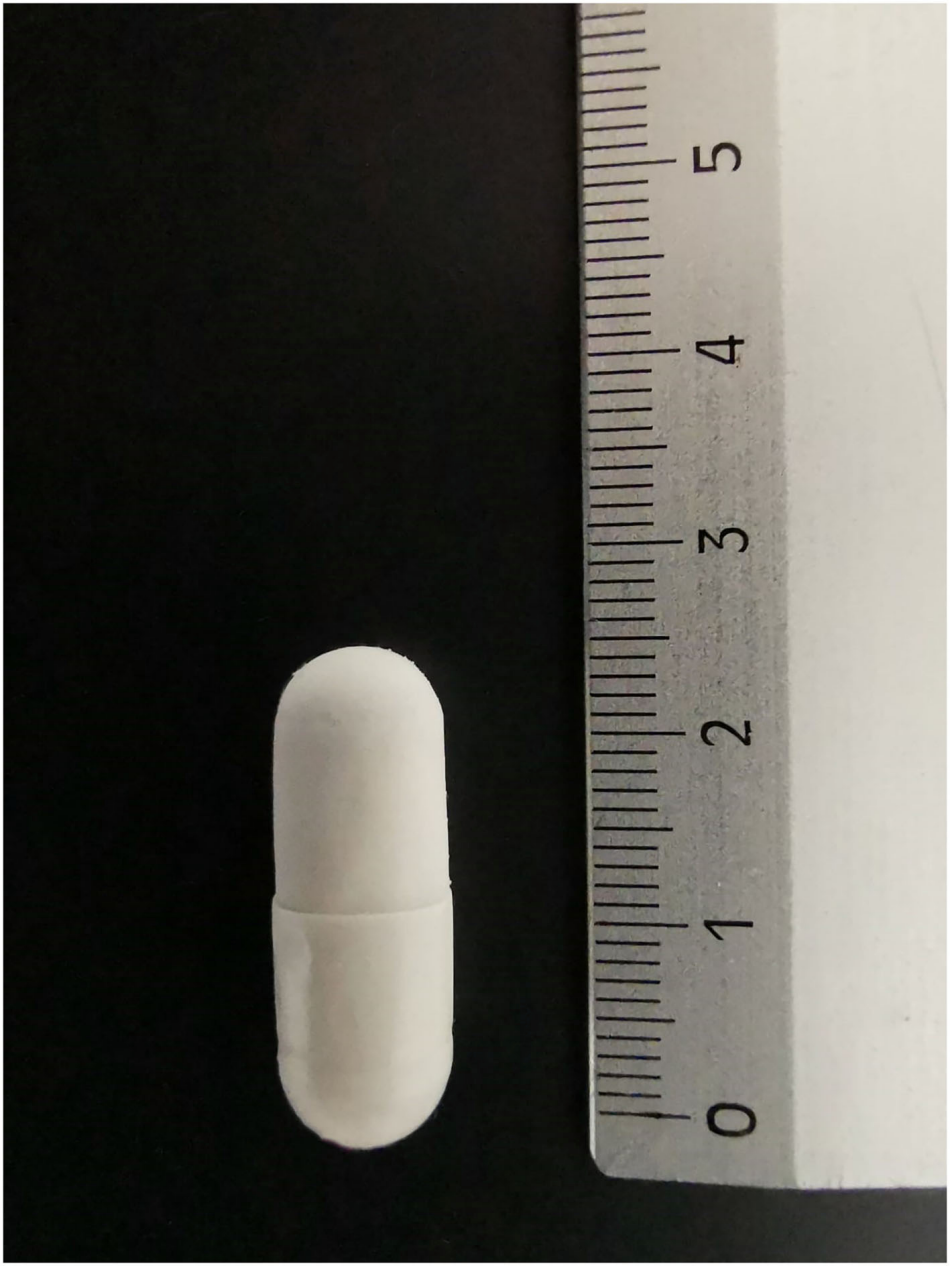
Example of capsule containing the fecal material (the reference in the ruler is in centimeters).

Fecal microbiota transplantation was performed in the form of frozen oral capsules (Figure 1); five capsules/10 kg of weight for five consecutive days [from T1 (day 1) to T5 (day 5)] were administered. Clinical monitoring was performed at T0 (the day before the first FMT = T1), at T5 (the last day of FMT), at T12 (1 week after the end of FMT = T5), and at T21 (16 days after the end of FMT = T5) (Table 1). The dog was also successively periodically monitored for a period of approximatively 1 year following FMT. The treatment was undertaken under the owner's signed informed consent, with a protocol approval issued from the Ethics Committee of the University of Perugia (Approval Number: 2018-23).

**Table 1:** Evolution of the canine inflammatory bowel disease activity index (CIBDAI) score of the patient after treatment.

	**CIBDAI variables**						**Total CIBDAI**
	**Attitude/activity**	**Appetite**	**Vomiting**	**Stool consistency**	**Stool frequency**	**Weight loos**	
**T0**	0	0	0	1	3	0	**4**
**T5**	0	0	0	1	2	0	**3**
**T12**	0	0	0	1	2	0	**3**
**T21**	0	0	0	1	2	0	**3**

*Fecal microbiota transplantation (FMT) was administered for 5 consecutive days, from T1 (day 1) to T5 (day 5)*.

### Clinical Outcome and Follow-Up

As previously described, at the time of FMT, the dog was presenting a CIBDAI score of four, with recurrent bloating and episodes of painful defecation. During the period of strict monitoring, 21 days in total, the last two conditions rapidly improved, with the latter being completely resolved, as reported by the owners, although not measured objectively. With regard to the CIBDAI score, it rapidly passed to a value of three (clinically insignificant disease—uniquely due to a reduction of the stool frequency score from 3 to 2) at T5 and remained the same (2) at T12 and T21 (Table 1). The body weight fluctuated between 29.8 and 30.5 kg. The owner also reported an improvement in “general” condition, with the patient appearing as “healthier” (non-objective evaluations). In the 18 months following FMT, the dog had some relapses that the owners defined as milder than before the FMT, as witnessed by the fact that during such periods, they never had to increase the prednisolone dose; such relapses were managed by temporarily slightly modifying the diet and with cycles of probiotics. During such periods, the dog underwent a small, programmed surgery (castration plus excision of a small subcutaneous nodule) followed by a short period of antibiotics resulting in negligible clinical effects on the GI tract. Around one and a half years after FMT, subsequent to an attempt to suspend prednisolone, the patient experienced a clinical relapse that needed a return to their use with, for a short period, a dose higher than the “maintenance” dose. No adverse effects associated with FMT were ever reported by the owners.

## Discussion

In a recent survey aiming at collecting information on the usage of FMT in dogs, it was found that it is mainly used for chronic enteropathies (64%) and then in acute diarrheic forms (36%), including parvovirosis (5). Similarly, previous literature reports its usage in dogs and cats in around 53% of cases in chronic enteropathies (including refractory *Clostridium perfringens*–associated diarrhea) and in 47% of acute diarrheas (7–24).

In this case report, we describe the use of FMT in one dog presenting with chronic relapsing diarrhea, needing a continuous low prednisolone dose to maintain the condition under acceptable control, however not preventing it from periodic relapses.

With regard to the route of administration, in the survey previously reported (5), it is described that the most common route of administration in dogs is through enemas, while very rarely (2 cases) through oral capsules. Then, taking into consideration the formerly published articles, it is very interesting to notice that only three out of 18 clinical studies/case series/case reports describe the administration of FMT through oral capsules (12, 15, 19). This report is therefore one of the very few describing the effects of FMT administered in the form of oral capsules.

Additionally, with regard to follow-up, the case described herein is unusual as we followed the case until around 18 months after administration, aiming at evaluating the short- and mid-term adverse effects as well as the short- and mid-term efficacy. Looking at previous literature, only two reports (1 in a dog and 1 in a cat) reached a follow-up time next to 1 year after treatment (in case of multiple treatments, reference is made to the last one performed) (8, 11). In our case, the follow-up ended when, due to the positive evolution of the condition, an attempt to reduce/suspend prednisolone was made; unfortunately, afterwards, a return to prednisolone administration was needed, as a clinical relapse was noticed as previously reported.

Regarding the possible steroid sparing effect of FMT in chronic enteropathies, the third aim of our evaluation, the comparison of our findings with already published articles has been quite difficult due to significantly different study designs; looking at some of them, a possible effect on reducing/discontinuing immunosuppressants, or in non-responsive enteropathies, can however be inferred (11, 15, 22, 24). In our case, neither during the 21 days of strict control nor during the following 18 months was it possible to reduce/suspend prednisolone, but what is noteworthy is that the milder relapses that the dog experienced after FMT never needed prednisolone dose increases as usually happened previously.

Finally, with regard to FMT's safety and efficacy in veterinary medicine, both aspects are generally considered positively (7–24). Similarly, in the case described herein, neither short- nor mid-term (1.5 year) adverse effects were noted and a reduction of the CIBDAI score, as well as of bloating and episodes of painful defecation, was observed.

The main limitation of this report is that we did not have the opportunity to associate microbiome, metabolome, and/or fecal proteome evaluations that could have helped to better understand the evolution of the intestinal environment.

In conclusion, this case report provides further evidence on the use of FMT in dogs suffering from relapsing chronic diarrhea; a very promising therapeutic approach that, however, in veterinary medicine still presents many aspects to be clarified and agreements to be found. In our case, FMT administered though oral capsules proved safe and effective by reducing the CIBDAI score and improving the general condition, and possibly decreasing the frequency of relapses and, therefore, avoiding the periodic increases in prednisolone necessary to keep them under control. Finally, the follow-up time reported herein is, moreover, to the best of our knowledge, the longest ever reported in dogs.

## Data Availability Statement

The original contributions presented in the study are included in the article/supplementary material, further inquiries can be directed to the corresponding author.

## Ethics Statement

The animal study was reviewed and approved by Ethics Committee of the University of Perugia (Approval Number: 2018-23). Written informed consent was obtained from the owners for the participation of their animal in this study.

## Author Contributions

MC, MTM, MI, FP, and FR contributed to conception and design of the study. MC, AM, and GR contributed to the acquisition, analysis, and interpretation of data for the work. MC and AM wrote the first draft of the manuscript. GR, MTM, MI, FP, and FR revised it critically for important intellectual content. All authors contributed to manuscript revision, read, and approved the submitted version.

## Conflict of Interest

The authors declare that the research was conducted in the absence of any commercial or financial relationships that could be construed as a potential conflict of interest.

## Publisher's Note

All claims expressed in this article are solely those of the authors and do not necessarily represent those of their affiliated organizations, or those of the publisher, the editors and the reviewers. Any product that may be evaluated in this article, or claim that may be made by its manufacturer, is not guaranteed or endorsed by the publisher.
